# Editorial: Advances in the Understanding of the Commensal Eukaryota and Viruses of the Herbivore Gut

**DOI:** 10.3389/fmicb.2021.619287

**Published:** 2021-03-02

**Authors:** Rosalind A. Gilbert, Sumit S. Dagar, Sandra Kittelmann, Joan E. Edwards

**Affiliations:** ^1^Department of Agriculture and Fisheries, Brisbane, QLD, Australia; ^2^Queensland Alliance for Agriculture and Food Innovation, The University of Queensland, Brisbane, QLD, Australia; ^3^Bioenergy Group, Agharkar Research Institute, Pune, India; ^4^Wilmar International Limited, WIL@NUS Corporate Laboratory, National University of Singapore, Singapore, Singapore; ^5^Laboratory of Microbiology, Wageningen University & Research, Wageningen, Netherlands

**Keywords:** rumen, anaerobic fungi, protozoa, phage, ecology, taxonomy, physiology

Herbivores play an important role in the survival of humanity, contributing food and textiles, as well as social and economic value. For decades, optimizing the productivity, health, welfare, and environmental footprint of herbivorous animals, particularly ruminant livestock, has been the subject of an extensive, global research effort. Much of this research effort has focused on the herbivore gut. The specialized nature of the herbivore digestive tract and its resident microbes enables the breakdown of highly fibrous plant materials, which are unable to be utilized by omnivores and carnivores. In recent years, the bacteria and methanogenic archaea have been the major focus of research efforts, with the other gut microbes being understudied in comparison.

The eukaryotic anaerobic fungi and ciliate protozoa represent up to half of the herbivore gut microbial biomass. They are generally recognized as mutualistic symbionts in ruminant animals where they have been most extensively studied to date. These eukaryotic microbes produce a wide range of highly potent, cellulolytic, hemicellulolytic, and amylolytic enzymes, which play a key role in feed degradation for the host. This is particularly true of anaerobic fungi, which are the most powerful fiber degraders in the known biological world. These specialist fungi initiate a physical and enzymatic attack on plant fiber that benefits the microbial community as a whole, not only by degrading highly complex carbohydrates but also by increasing the accessibility of substrates and facilitating biofilm formation. In addition, due to the production of hydrogen in their hydrogenosomes, anaerobic fungi and ciliate protozoa provide micro-habitats for hydrogen-scavenging bacteria and methanogenic archaea.

The herbivore gut microbiome also contains a dense and diverse population of viruses. The majority of these viruses actively infect and replicate within the microbes resident in the gut (for example, bacteriophages and archaeal viruses), and viral genomes (prophages) can often be found integrated into the genomes of gut microbes. Viruses contribute to gene transfer and cause microbial lysis, resulting in the release of microbial enzymes and modulation of microbial community diversity.

Despite the clear importance of the anaerobic fungi, ciliate protozoa, and viruses to our understanding of herbivore gut function, only bacteria and archaea are commonly characterized in the majority of herbivore gut studies. This Research Topic, therefore, has focused on reviewing current knowledge and reporting original research and technical advances in our understanding of the roles of commensal eukaryotes (anaerobic fungi and ciliate protozoa) and viruses in the herbivore gut.

Historical and current research, along with future perspectives, were detailed in three comprehensive reviews focused on anaerobic fungi (Hess et al.), ciliate protozoa (Firkins et al.) and viruses (Gilbert et al.). Each of these reviews represents a collaborative effort, drawing on contributions from multiple international research groups. Therefore, they provide a unique snapshot of collective knowledge for each of the respective microbial taxa. These reviews also provide a valuable learning resource, although of particular note is the extensive image library of rumen protozoa (https://ansci.osu.edu/our-people/jeffrey-l-firkins). This library was collated by the laboratory of Burk Dehority and has been made available electronically to aid researchers in understanding protozoal morphology for evaluation and identification purposes (Firkins et al.).

Interactions between the anaerobic fungus *Pecoramyces* sp. F1 and methanogenic archaea were comprehensively investigated using multiple molecular-based approaches (genomic, transcriptomic and proteomic) by Li et al. These tools allowed the authors to provide a detailed mechanistic understanding of the metabolism of the anaerobic fungus-methanogen syntrophic co-culture.

Metatranscriptomics and protein expression techniques were used to show that rumen protozoa produce several carbohydrate-active enzymes (i.e., glycosyl hydrolases 5 and 11, polysaccharide lyases, deacetylases, and xylanases) as well as enzymes active against pectin, mannan and chitin (Williams et al.). Therefore, this study highlighted the predatory capacity of ruminal protozoa as well as the significant contribution these eukaryotes make to carbohydrate breakdown and fermentation in the rumen.

A significant gap in current knowledge of gut viruses was addressed by the isolation and sequencing of novel viruses (phages) infecting the rumen bacterial genus *Butyrivibrio* (Friedersdorff et al.). In addition to this new fundamental knowledge, the addition of sequences for rumen-sourced phage isolates to publicly available sequence databases represents an important and essential step forward for the research community. This will increase the accuracy of viral gene taxonomic and functional annotation, facilitating future advances in the understanding of gut viral communities.

Original research and technical perspectives were provided by two studies investigating the use of alternative techniques for determining the taxonomy and ecology of anaerobic fungal (Edwards et al.) and protozoal communities (Cedrola et al.) in the herbivore gut. Both these studies provided guidance for researchers unfamiliar with the technologies used, and highlighted the challenges and opportunities associated with the study of these specialist eukaryote communities.

As well as reviewing and advancing our understanding of eukaryota and viruses in the herbivore gut, the compilation of the published articles in this e-book, highlights the significant and inspirational efforts made by several research groups, particularly those who focused on the early studies of anaerobic fungi [Professor Colin Orpin and the late Professor Anthony (Tony) Trinci] and protozoa (the late Professor Burk Dehority). The ground-breaking work of Colin Orpin in the early 1970's was a paradigm shift in the understanding of fungal biology, as until his work, all fungi were believed to respire aerobically (Orpin, 1975, 1984; Mountfort and Orpin, 1994). From a long career commencing in the 1960's, Tony Trinci also significantly contributed to current understanding of both anaerobic fungi and mycology in general, exploring fungal physiology, enzymology and providing the conceptual foundations of fungal multicellular growth (Trinci, 1974; Lowe et al., 1987; Trinci et al., 1994). Between 1957 and 2013, Burk Dehority pioneered methodology and research into the synergism of prokaryotic and eukaryotic microbial species for the digestion of plant fiber (Dehority, 1984, 1993, 2003). He shed light onto the vast diversity, function, and beauty of ciliate protozoa, in both domesticated and non-domesticated herbivorous animals, and described no less than 21 new species.

While many historical, current and new advances are detailed in this e-book, collectively this body of work emphasizes the urgent need to adopt more holistic approaches to the study of the herbivore gut microbiome. Only then can the complex interactions within the herbivore gut microbiome be better understood, facilitating the development of novel and sustainable approaches to benefit the nutrition and health of the host animal, whilst mitigating the ecological impact and environmental footprint of livestock-based agriculture.

## Author Contributions

All authors listed have made a substantial, direct and intellectual contribution to the work, and approved it for publication.

## Conflict of Interest

SK is an employee with Wilmar International Limited. The remaining authors declare that the research was conducted in the absence of any commercial or financial relationships that could be construed as a potential conflict of interest.
